# 

**Published:** 1881-09

**Authors:** 


ANNOUNCEMENT.—We will organize for the INDEPENDENT PRACTITIONER an editorial staff composed of prominent and able gentlemen, some of whom may be selected from other places than the city of New York. The corps shall represent medical, surgical, obstetrical, dental, hygienical and popular sciences, and in these we will search for that which is new and useful and give the refined findings to our readers. Politics, religious creeds and isms or preferences in colleges and cliques shall be excluded, and we will uphold right, truth and justice with independence. We shall endeavor to build an arch the keystone of which shall be that of *science and truth*, on which men of all nations and all creeds shall be able to halt as they pass over, not amid "thorns and thistles," but where they can read a refreshing truth which may encourage them through the journey of life.

To further this end we solicit original matter for our columns from those who approve of our plan, and subscriptions from all. We trust that our future may be improved by the experience of our many errors in the past, that all short-comings be forgiven if not forgotten, and that our contributors and subscribers will accept our sincere thanks for their continued favors, and we hope they will induce others, in our behalf, to follow their generous example.

For the *present* please send all mail matter *for the editors*, such as exchanges and articles for publication, to 68 N. Charles street, Baltimore, Md., and all subscriptions, advertisements and business matters of all kinds belonging to the journal, to No. 20 Courtlandt street, N. Y. BASIL M. WILKERSON, Editor and Proprietor.

WILL *YOU* PLEASE SEND IN *YOUR* SUBSCRIPTION MONEY *NOW!*

MANUFACTURING CHEMISTS.

Money is at OUR RISK ONLY when sent by Post Office Money Order, Registered Letter, or Check on Banks in New York City, made payable to INDEPENDENT PRACTITIONER, 20 Courtlandt street, N. Y. City.

